# Factors associated with smoking in immigrants from non-western to western countries – what role does acculturation play? A systematic review

**DOI:** 10.1186/s12971-015-0036-9

**Published:** 2015-04-16

**Authors:** Katharina Reiss, Jessica Lehnhardt, Oliver Razum

**Affiliations:** Department of Epidemiology & International Public Health, Bielefeld School of Public Health (BiSPH), Bielefeld University, P.O. Box 10 01 31, 33501 Bielefeld, Germany

**Keywords:** Systematic review, Immigrants, Smoking, Acculturation, Smoking epidemic

## Abstract

**Introduction:**

We aimed to identify factors associated with smoking among immigrants. In particular, we investigated the relationship between acculturation and smoking, taking into consideration the stage of the ‘smoking epidemic’ in the countries of origin and host countries of the immigrants.

**Methods:**

We searched PubMed for peer-reviewed quantitative studies. Studies were included if they focused on smoking among adult immigrants (foreign-born) from non-western countries now residing in the USA, Canada, Ireland, Germany, the Netherlands, Norway, the UK, and Australia. Studies were excluded if, among others, a distinction between immigrants and their (native-born) offspring was not made.

**Results:**

We retrieved 27 studies published between 1998 and 2013. 21 of the 27 studies focused on acculturation (using bidimensional multi-item scales particularly designed for the immigrant group under study and/or proxy measures such as language proficiency or length of stay in host country) and 16 of those found clear differences between men and women: whereas more acculturated women were more likely to smoke than less acculturated women, the contrary was observed among men.

**Conclusion:**

Immigrants’ countries of origin and host countries have reached different stages of the ‘smoking epidemic’ where, in addition, smoking among women lags behind that in men. Immigrants might ‘move’ between the stages as (I) the (non-western) countries of origin tend to be in the early phase, (II) the (western) host countries more in the advanced phase of the epidemic and (III) the arrival in the host countries initiates the acculturation process. This could explain the ‘imported’ high (men)/low (women) prevalence among less acculturated immigrants. The low (men)/high (women) prevalence among more acculturated immigrants indicates an adaptation towards the social norms of the host countries with ongoing acculturation.

## Introduction

High-income or economically developed western countries, such as North America, North and West Europe, Australia, and New Zealand are characterised by considerable ethnic diversity. The immigrant populations residing in western societies are heterogeneous, comprising people from different countries of origin, with different motivations to migrate, cultural identities etc. Health outcomes among immigrants in terms of mortality and morbidity have been largely covered in international research. Fewer studies focused on immigrants’ health behaviour and especially on the determinants of health behaviour such as smoking. Smoking is one of the leading causes for premature death and thus a particularly risky health behaviour. It causes a variety of cancers and cardiovascular diseases [1-4]. On the individual level, smoking patterns vary with socioeconomic status – and here especially with education – with stress, age, and gender. Smoking is also influenced by peer group social norms on the interpersonal level and country-specific tobacco control interventions (public and work place bans, cigarette prices etc.) or the society’s social support system of smoking on the socio-political level [5-15].

**Figure 1:**
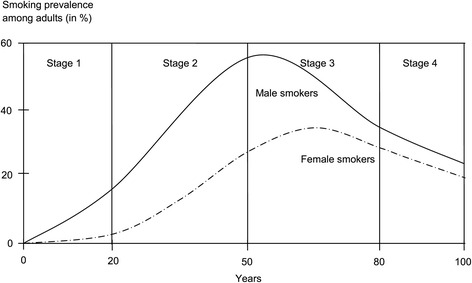
The stages of the ‘smoking epidemic’ proposed by Lopez et al. in 1994.

Smoking behaviour as well as norms and attitudes towards smoking also differ between countries. Lopez et al. [16] speak of the ‘smoking epidemic’, a four-stage model describing the progress of smoking among men and women: in the 1^st^ stage smoking predominantly involves men. In the 2^nd^ stage, smoking prevalence increases steeply among men and slightly among women. The 3^rd^ stage is characterised by a further increase in smoking among women. Smoking among men starts declining and prevalences among men and women converge. The 4^th^ stage shows a further decline in prevalence among both men and women (see Figure 1). Rather than looking at the stages as isolated parts, the model should be understood as a process or continuum over decades [17]. Whereas economically developed western countries are in the advanced phases of the epidemic, countries such as China, India, Syria or some Southeast Asian or African countries are more located towards the beginning or early phases of the epidemic [18-22].

Thus, immigrants from non-western to western countries move from an earlier to a more advanced stage of the epidemic. As health risks and resources they acquired in their countries of origin are not static and may be subject to change in the host countries, immigrants might adapt to, for example, the host country’s smoking behaviour [23]. This change in smoking behaviour might be the result of an acculturation process which starts immediately after arrival in the host country. Acculturation is a complex phenomenon: it refers to a dynamic process through which behaviours of immigrants change as a result of interactions with individuals in their (new) social and cultural environment [24]. The concept of acculturation can be either understood as unidimensional – where immigrants move along a continuum ranging from a weak adaptation to the host culture to a strong one – or as bidimensional – where immigrants may independently maintain the culture of origin and adapt to the host culture. The best known bidimensional acculturation model was developed by Berry [25], although recent research activities propose a more extensive approach to acculturation. Schwartz et al. [26], for example, also incorporate cultural practices, such as customs and traditions, cultural values, such as individualism or independence and a cultural identification, such as the attachment to a certain cultural group. It becomes apparent that acculturation is influenced by three main contextual areas: the context prior to immigration, the immigration context itself and the settlement context [27].

Acculturation models are widely used in health behaviour research with various measures, ranging from indirect proxy measures (e.g. time spent in host country, or proficiency in the language of the host country), to multi-item scales [27-30]. The relationship between acculturation and, for example, substance abuse, dietary practices, leisure-time activity or health services use has mainly been studied in the United States [31-38]. There is also evidence from the US linking smoking behaviour to level of acculturation [39-45]. In Europe, research on this relationship is scarce – smoking among immigrants has been investigated mainly with regard to the association between smoking and socioeconomic status.

**Figure 2:**
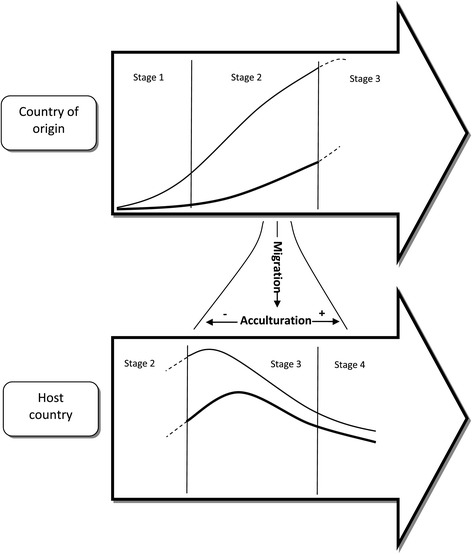
Schematic diagram on the association between the ‘smoking epidemic’ and acculturation.

A combination of the ‘smoking epidemic’ model and the acculturation model should allow to predict the smoking behaviour of immigrants: populations that migrate from non-western to western countries will ‘bring along’ the smoking behaviour from their countries of origin and maintain it for some time after migration. With the commencing acculturation process in the host countries, these ‘imported’ risks will change and immigrants will adapt to the smoking behaviour of the majority population in the host country (see Figure 2). This assumes that acculturation is an important (albeit not necessarily the only) determinant of smoking behaviour among immigrants.

The aim of this paper is to identify common patterns for smoking behaviour among immigrants (or foreign-born persons) from non-western to western countries with a special focus on the role of acculturation through a systematic review over the international literature.

## Methods

### Search strategy

**Figure 3:**
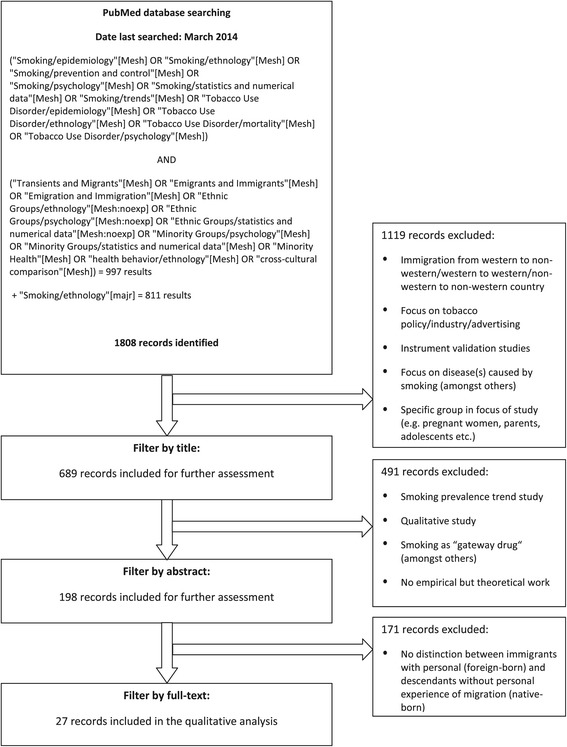
Inclusion and exclusion of publications in the systematic review.

The database PubMed was searched in May 2012 and again in March 2014 using the MeSH terms displayed in Figure 3. All studies were filtered first by title and then by a narrower filter procedure following the inclusion and exclusion criteria listed below. Next, all full-texts of the remaining articles were read and then entered for the review or dropped. Additionally, the reference lists of included articles were scanned for studies that were not detected by the database search.

### Eligibility criteria

All peer-reviewed primary studies in English language published after 1990 were included. Studies were included if they matched the following criteria: quantitative study; analysis of factors associated with cigarette smoking (irrespective of frequency and quantity); focus on immigrant adults (18 years and older); specification of country of origin; personal migration experience from an economically developing non-western country of origin to an economically developed western host country (North America, North and West Europe, Australia, and New Zealand). Studies that focused on foreign- and native-born persons were included if the proportion of immigrants or foreign-born persons was more than 90%. Studies with less than 90% were only included if a clear distinction between foreign- and native-born persons was made and factors associated with smoking were examined and presented separately for each group.

Studies were excluded if no distinction between immigrants (foreign-born) and their offspring (born in the host country) was made, if the immigrant group under study was defined by a specific characteristic (e.g. pregnancy, occupation, addiction etc.); if the study focused on risk factors for (or causes of) certain diseases, was not empirical but theoretical, merely investigated time trends in smoking prevalence, focused on any form of drug intake or any form of tobacco use other than smoking cigarettes, was conducted for validating a questionnaire, evaluated a prevention programme or focused on media/advertisement influence on smoking behaviour.

### Synthesis of results

The results reported in the articles were included as *findings* in this review if they were derived from any form of statistical analysis, regardless of whether descriptive analyses or regression models. For example, variables which were not significantly associated with smoking in simple regression analyses might have not been included in the subsequent multiple regression analyses. As non-significance does not equate with non-relevance, all factors known to be relevant were covered in this review. In the case of conflicting results between descriptive and analytical procedures, those derived from the highest level of statistical analysis were counted as finding. A meta-analysis was not performed as definitions and measurements of the variables of interest were not consistent between the studies.

The retrieval procedure was performed independently by two investigators. By the end of the first retrieval round, there were five articles with conflicting decisions regarding inclusion. After discussion, four articles were excluded and one was included in the review. After the second search there were no discrepancies. The final number of studies included was 27. The retrieval procedure and the criteria for exclusion and inclusion are outlined in Figure 3.

The two investigators also independently assessed and documented methodological quality of the studies to trace possible bias within as well as across the studies. The following information was documented for each study: aim, definition of the immigrant group, data source (if secondary data was used) or sampling procedure (if primary data was generated), method of data collection, study design, number of immigrant participants, statistical methods applied, operational definition of dependent and independent variables, and limitations reported.

## Results

### Study characteristics

The 27 studies included were published between 1998 and 2013 and all of them used a cross-sectional design; the majority (19 of 27) were conducted in the USA; two were conducted in Germany, and one each in Ireland, Canada, the Netherlands, the UK, Norway, and Australia. Most of the studies focused on Asian immigrants, originating from China (5 of 27), Korea (3 of 27), Vietnam (2 of 27), and the Philippines (1 of 27). Three studies investigated more than one Asian immigrant group. The second-largest immigrant group under study were Latinos/Hispanics from Mexico (2 of 27) and El Salvador (1 of 27). One study focused on African immigrants. One study compared the smoking behaviour between Asians and Latinos/Hispanics among others. Two studies focused on Arabs. One study each investigated smoking among Ethiopians, Polish immigrants, Turkish immigrants, and immigrants from the former Soviet Union; and two studies focused on more than one immigrant group. The sample sizes ranged from 96 to 16,738 persons. Eleven studies had a sample size below 1,000, in ten studies the sample size ranged from 1,000-5,000 participants and six studies surveyed more than 5,000 persons. Fifteen of 27 studies focused on foreign-born persons only, in seven studies more than 90% were foreign-born, in five studies between 7% and 77% of the participants were foreign-born. Four studies restricted their study sample to men and five studies performed their highest statistical analysis only for men as the number of female smokers was too small. Eleven of 27 studies stratified their analysis by gender, seven adjusted for gender.

### Study findings

Table 1 presents contextual information of the different countries, such as the GDP per capita as prosperity indicator and the smoking prevalence in the immigrants’ host countries, their countries of origin and among immigrants themselves as reported by the studies included: smoking prevalence among men in the countries of origin is higher than that among men in immigrants’ host countries, whereas the contrary applies to women. Additionally, the gap in smoking prevalence between men and women is larger in the countries of origin than in the host countries. Among immigrants, this gender-difference is not as large as in their countries of origin but still larger than in the host countries.

**Table 1:** **Comparison of Gross Domestic Product (GDP) per capita (US$), and smoking prevalence in countries of origin, host countries and among immigrants**

**Country of origin of immigrants**	**GDP per capita (US$, 2012) in country of origin** ^**1**^	**Smoking prevalence in country of origin in 2011** ^**2**^	**Smoking prevalence among immigrants according to studies included in the review**	**Study reporting the smoking prevalence among immigrants** ^**7**^
**Host country: United States of America | GDP per capita (US$, 2012): 51,749 | Smoking prevalence (2011): Men 21%, Women 17%**				
China	6,091	Men: 47%	Men: 16%	2
		Women: 2%	Women: 7%	
			Men: 14%	7
			Women: 6%	
			Men: 18%	11
			Women: 4%	
			Men: 25%	12
			Women: 3%	
			Men: 15%	6
			Women: 4%	
			Men: 34%	15
			Women:2%	
			Total: 24%	14
			Hmong^4^ men: 12%	1
			Hmong^4^ women: 1%	
Cambodia	945	Men: 42%	Men: 14%	1
		Women: 3%	Women: 2%	
			Total: 42%	14
Laos	1,412	Men: 48%	Men: 32%	1
		Women: 4%	Women: 4%	
Republic of Korea	22,590	Men: 49%	Men: 32%	10
		Women: 8%	Women: 4%	
			Men: 39%	16
			Women: 7%	
			Men: 37%	6
			Women: 9%	
			Men: 27%	13
			Total: 27%	14
Vietnam	1,755	Men: 46%	Men: 42%	1
		Women: 2%	Women: 1%	
			Men: 32%	6
			Women: 2%	
			Men: 32%	8
			Men: 43%	19
			Total: 40%	14
Philippines	2,587	Men: 44%	Men: 35%	5
		Women: 10%	Men: 24%	7
			Women: 10%	
			Men: 25%	6
			Women: 8%	
Mexico	9,749	Men: 27%	Men: 33%	4
		Women: 8%	Women: 11%	
			Men: 29%	9
			Women: 10%	
			Hispanic^5^ men: 20%	7
			Hispanic^5^ women: 8%	
El Salvador	3,790	Men: 24%	Men: 20%	17
		Women: 3%	Women: 3%	
			Hispanic^5^ men: 20%	7
			Hispanic^5^ women: 8%	
Sub-Saharan Africa^3^	1,417	Men: 22%	Men: 22%	18
		Women: 4%	Women: 7%	
Arab world^3^	7,048	Men: 37%	Men: 60%	3
		Women: 4%	Women: 11%	
South Asia^3^	1,398	Men: 36%	Men: 16%	6
		Women: 8%	Women: 3%	
**Host country: Canada GDP per capita (US$, 2012): 52,219 Smoking prevalence (2011): Men 20%, Women: 15%**				
Ethiopia	454	Men: 8%	Men: 28%	20
		Women: <1%	Women: 10%	
**Host country: Germany GDP per capita (US$, 2012): 41,863 Smoking prevalence (2011): Men 35%, Women: 25%**				
Former Soviet Union^3^	9,464	Men: 44%	Men: 37%	22
		Women: 11%	Women: 16%	
Turkey	10,666	Men: 42%	Men: 49%	23
		Women: 13%	Women: 28%	
**Host country: Ireland** ^**6**^ **GDP per capita (US$, 2012): 45,932 Smoking prevalence (2011): Men 23%, Women: 20%**				
Poland	12,708	Men: 38%	Men: 51%	21
		Women: 27%	Women: 40%	
**Host country: Australia GDP per capita (US$, 2012): 67,556 Smoking prevalence (2011): Men 21%, Women: 19%**				
Arab world^3^	7,048	Men: 37%	Men: 44%	27
		Women: 4%	Women: 23%	
**Host country: The Netherlands GDP per capita (US$, 2012): 45,955 Smoking prevalence (2011): Men 29%, Women: 23%**				
Turkey	10,666	Men: 42%	Men: 63%	24
		Women: 13%	Women: 32%	
Morocco	2,902	Men: 32%	Men: 30%	24
		Women: 2%	Women: 1%	
Suriname	9,376	Men: 17%	Men: 55%	24
		Women: 3%	Women: 30%	
**Host country: Norway GDP per capita (US$, 2012): 99,558 Smoking prevalence (2011): Men 28%, Women: 26%**				
Turkey	10,666	Men: 42%	Men: 56%	25
		Women: 13%	Women: 28%	
Iran	6,816	Men: 26%	Men: 42%	25
		Women: 1%	Women: 23%	
Pakistan	1,257	Men: 38%	Men: 34%	25
		Women: 7%	Women: 4%	
Vietnam	1,755	Men: 46%	Men: 36%	25
		Women: 2%	Women: 5%	
Sri Lanka	2,923	Men: 31%	Men: 19%	25
		Women: 1%	Women: 1%	
**Host country: United Kingdom | GDP per capita (US$, 2012): 39,093 | Smoking prevalence (2011): Men 22%, Women: 22%**				
China	6,091	Men: 47%	Men: 24%	26
		Women: 2%	Women: 1%	

^1^Source: The World Bank – GDP per capita (currency US$) 2012. URL: http://data.worldbank.org/indicator/NY.GDP.PCAP.CD.

^2^Source: WHO Report on the Global Tobacco Epidemic from 2013 (Ethiopia: 2011, Suriname: 2009). URL: http://www.who.int/tobacco/global_report/en/.

^3^As no measures are available for these aggregated geographical areas, GDP per capita was calculated as sum of GDP divided by sum of population of all countries, smoking prevalences were averaged across all countries.

^4^Hmong people originate from many different Southeast Asian and East Asian countries. As most of them live in China, prevalences were assigned to China.

^5^Hispanic or Latino Americans originate from different Latin American countries or the Iberian Peninsula. As only immigrants from Mexico and El Salvador were investigated in the scope of this review, prevalences were assigned to each of both countries.

^6^Source: Health Service Executive: Cigarette Smoking Prevalence in Ireland 2013. URL: http://www.hse.ie/eng/about/Who/TobaccoControl/Research/.

^7^Studies are presented as numbers, further information on studies is specified in Table 4.

Table 2 presents the factors associated with smoking among immigrants, for men and women combined if analyses were only adjusted for gender or no gender-specific differences were observed; or for men and women separately if gender-specific differences were observed or analyses were restricted to one gender only.

**Table 2:** **Findings of the systematic review**

**Factors associated with smoking**		**Smoking was positively associated with the stated factor among**			**Studies reporting the association** ^**#**^
		**Men and women**	**Men**	**Women**	
**Acculturation scale**		Low vs. high			3
		High vs. low			9
			Low vs. high	High vs. low	1,10,12*
**Acculturation with main focus on the following proxy measures:**	**Proficiency in the language of host country**		Low vs. high	High vs. low	11*,7,5*,6, 8*,19*
	**Length of stay in host country**		Short vs. long	Long vs. short	13*,6
	**Country of birth** ^**$**^		Foreign-born vs. native-born	Native-born vs. foreign-born	7
	**Country of smoking initiation**	Country of origin vs. host			4,9
	**Age at migration**	Young vs. old			9
**Proficiency in the language of host country** ^**§**^			Low vs. high	High vs. low	15*,16*
**Length of stay in host country** ^**§**^		Long vs. short			18,21
			Short vs. long	Long vs. short	2*,16*,20,22,23
**Country of birth** ^**§,$**^		Native-born vs. foreign-born			18,23
**Gender**		Being male vs. female			All studies except for 5*,8*,13*,19*
**Educational level**		Low vs. high			1,20,21,18,14,7,6,27
			Low vs. high	High vs. low	19*,5*,15*,16*, 2*,8*,4,22,23,24,25
**Employment**		No vs. yes			18,27
		Yes vs. no			21,10
			Yes vs. no		11*,13*
**Age**		Young vs. old			1,14,10,26,27
			Young vs. old		2*,13*,11*,19*
**Income**		Low vs. high			7,18
			Low vs. high		5*,2*
**Religion**			Not religious vs. religious		20,16*
**Marital status**		Non-married vs. married			20,14,7,18,6,27
			Non-married vs. married		8*
**Self-assessed health status**		Poor vs. good			27
**Pre-migration life events**			Exposed vs. non-exposed	Non-exposed vs. exposed	20
**Post-migration life events**			No association	Exposed vs. non-exposed	20
**Social support**			Satisfied vs. not satisfied	No association	20
**Alcohol consumption**		Yes vs. no			6
			Yes vs. no		13*,8*,16*
**Knowledge of tobacco risks**			Low vs. high/moderate		11*
**Knowledge of cancer warning signs**			No vs. yes		15*
**Perceived risk of smoking-related diseases**			High vs. low		5*
**Blood check and/or physical checkup**			No vs. yes		8*
**Source of health care**			Non-western vs. western		15*
**Role model smoking**			Yes vs. no		19*,5*
**Depression score**			Higher vs. lower		19*
**Regular exercise**			Less vs. more		19*
**Health insurance**			Not available vs. available		11*,19*
**Geographical area of origin (in country of origin)**			Rural vs. urban		19*

^#^Studies are presented as numbers, further information on studies is specified in Table 4.

*Indicates studies that either restricted their highest statistical method or the study population in general to men only.

^$^Only applicable to studies that investigated foreign- and native-born migrants.

^**§**^Variables that were included in the studies without linking them to acculturation. Thus, the respective variable is listed separate from acculturation.

Acculturation was focused on in 21 of all 27 studies. Table 3 illustrates the different acculturation measures used. Only five studies applied multi-item acculturation scales that were developed particularly for the immigrant group under study and were based on a bidimensional concept of acculturation. Multiple questions were used to identify the preference for and the fluency in the culture of the country of origin or the host country. Higher scores on the scales reflected an orientation more towards the host country culture. All other studies used proxy measures for acculturation, mainly proficiency in the language of the host country and length of stay. The majority of the studies (16 of 21) that included acculturation emphasised differences by gender, irrespective of the acculturation measure applied (acculturation scale or proxy measure for acculturation): less acculturated men (or men with lower language proficiency or short length of stay) had higher smoking prevalences than more acculturated men (or men with higher language proficiency or long length of stay). However, the contrary applied to women. Women with a lower level of acculturation (or lower language proficiency or short length of stay) had lower smoking prevalences than women with a higher acculturation level (or higher language proficiency or long length of stay). These gender-specific patterns were observed both in studies that explicitly stated that they used proxy variables to measure acculturation (11 of 21) and in studies that did not link variables such as length of stay or language proficiency to acculturation (5 of 21).

Gender was one of the factors most frequently reported to be associated with smoking behaviour in immigrants (23 of 27). The majority of the studies reported marked differences by gender, with men being more likely to be current smokers than women.

Additionally, educational level (19 of 27), age (9 of 27), employment (6 of 27), and marital status (7 of 27) were frequently reported. Younger and non-married persons were more likely to smoke than older and married persons. Smoking was positively associated with a low educational level among men but with a high educational level among women (11 of 27). In 4 out of 27 studies, employed persons had a higher smoking prevalence than unemployed persons. Other factors, such as income, religion, alcohol consumption or knowledge of tobacco health risks, were reported by less than five studies (see Table 2), and often only for men.

**Table 3:** **Acculturation measures used in the studies included for the review**

Study*	**Acculturation measure (proxy variable)**	**Were proxy measures explicitly used to measure acculturation?**	**Acculturation measure (scale)**
1	Primary language, percentage of life lived in USA	Yes	Community and acculturation identification measure (21 items); two main constructs: cultural fluency and cultural orientation (to assess country of origin or US identification)
2	Length of stay	No	
3			MAAS scale (Male Arab-American Acculturation) (8 items); two main constructs: separation and assimilation & integration and marginalisation
4	Country of smoking initiation	Yes	
5	Length of stay, self-perceived ethnic/cultural identity, language proficiency (English, Tagalog)	Yes	
6	English language used at home, length of stay	Yes	
7	English language proficiency, length of stay, country of birth	Yes	
8	English language proficiency, language used at home, language used with friends, length of stay	Yes	
9	Age at migration, country of birth, country of smoking initiation	Yes	Bidimensional Acculturation Scale for Hispanics (4 items)
10			The Suinn-Lew Asian self-identity acculturation to US society scale (education and length of stay added here)
11	English language used at home, reading of English newspapers	Yes	
12			Linguistic acculturation scale for Southeast Asians (7 items); two main constructs: English language proficiency, Chinese language proficiency
13	Length of stay	Yes	
14	Legal immigrant status, length of stay	No	
15	English language proficiency	No	
16	Length of stay, English language proficiency	No	
17	Length of stay, age at migration	Yes	
18	Length of stay, country of birth	No	
19	English language proficiency	Yes	
20	Length of stay	No	
21	Length of stay	No	
22	Length of stay	No	
23	Length of stay, country of birth	No	
24	Social contacts with the host population, years of immigration	Yes	
	→ only used to characterise the study population, no further consideration		
25	Years lived in host country before age of 16	No	
	→ only used to characterise the study population, no further consideration		
26	None		
27	None		

^*^Studies are presented as numbers, further information on studies is specified in Table 4.

### Quality assessment of the studies included for the review

All studies presented a clear study aim, defined their study population accordingly and clearly specified dependent and independent variables. Besides descriptive analyses, logistic regression analyses were applied in 20 of 27 studies (see Table 4).

**Table 4:** **Methodological quality assessment of the articles included in the review**

**No.**	**Authors**	**Year and country of study**	**Study aim**	**Immigrant group under study (definition of immigrants)**	**Data source (secondary data) or sampling method (primary data)**	**Data collection method**	**Study design**	**Number of immigrant participants**	**Operational definition of variables used (DV = dependent variable; IV = independent variable)**	**Statistical methods applied besides standard descriptive analyses**	**Reported study limitations**
1	Constantine, Rockwood, Schillo, Alesci, Foldes, Phan, Chhith, Saul [56]	2010 in USA	To explore relationship between smoking and acculturation	Hmong, Vietnamese, Cambodian and Laotian Americans (country of birth, self-identification)	Sample construction via list of surnames common to specific community, utilization of telephone screener for recruitment where a household member was selected at random	Interviewer- and telephone-administered survey (translated instrument, bilingual interviewers)	Cross-sectional	1,615 (95% foreign-born)	DV: current smoker vs. non-smoker IV: age, education, % of life lived in USA, acculturation	Logistic regression Stratified by gender, for males also by nativity	Self-reported tobacco use, underreporting of smoking among Vietnamese, Cambodian, and Lao populations possible, small sample size for within group (ethnicity and gender) analysis
2	Hu, Pallonen, Meshack [57]	2010 in USA	To analyse impact of immigration status on tobacco use	Chinese Americans (country of birth, country of residence before immigration)	Chinese-American households with listed residential telephone numbers and list of Chinese surnames, random household selection by stratified probability sampling method with geographically proportional allocation, additional public relations work	Self-administered (mail) and telephone-administered survey (translated instrument, bilingual interviewers)	Cross-sectional	1,054 (94% foreign-born)	DV: current smoker vs. non-smoker IV: age, income, education, years living in the USA	Logistic regression Highest statistical analysis only for males	Failure to reach two-thirds of all potential respondents, more than one-third of potential respondents in initial sampling frame denied they were Chinese, small sample size of female Chinese-American smokers
3	Al-Omari, Scheibmeir [58]	2009 in USA	To describe relationship between tobacco use and psychological acculturation	Arab American (country of birth)	Convenience-sampling method: 2 grocery stores and one Islamic center were used to recruit participants	Self-administered survey (instrument only in English, no information on presence of bilingual interviewers)	Cross-sectional	96 (100% foreign-born)	Nicotine dependence with tobacco exposure and acculturation (acculturation also by gender)	Pearson correlation statistics	Small number of participants and nonrandomised sampling, large overrepresentation of men in sample, instrument only available in English language
4	Stoddard [59]	2009 in USA	To examine impact of social and structural factors on risk of smoking initiation among immigrants before and after immigration	Mexican American (self-identification)	Data source: National Health Interview Survey (NHIS) (nationally representative survey in the USA conducted annually)	Interviewer-administered survey (translated instrument, bilingual interviewers)	Cross-sectional	6,935 (58% foreign-born)	DV: risk of regular smoking initiation IV: age, education, country of smoking initiation	Discrete-time hazard analysis Stratified by gender	Only limited analysis of social and structural factors, use of retrospective data on age at immigration and age of smoking initiation (recall bias due to cohort design possible), data on exact age at immigration were unavailable and had to be approximated or imputed
5	Maxwell, Garcia, Berman [60]	2007 in USA	To examine knowledge, beliefs and attitudes towards smoking and to examine relationship between i.e. duration of stay and acculturation with smoking	Male Filipino Americans (probably self-identification, not further specified)	Sampling by approach of community-based organizations serving Filipino Americans, Filipino American associations, Christian churches, businesses	Interviewer-administered survey (translated instrument, bilingual interviewers)	Cross-sectional	318 (100% foreign-born)	DV: current smoker vs. non-smoker IV: age, duration of residency in USA, English usage with friends, education, income, employment, health insurance, knowledge score, smoking beliefs score, perceived risk of lung cancer, perceived risk of smoking-related diseases, most friends smoke	Logistic regression	Community sampling, sample was restricted to Filipino men aged 40-75 years, cross-sectional design, assessment of some constructs with only single items to keep survey brief
6	An, Cochran, Mays, McCarthy [61]	2008 in USA	To estimate effects of multiple acculturation indicators on current smoking, to compare gender- and ethnic subgroup-specific current smoking prevalence, to examine effects of other potential predictors of smoking behavior for men and women	Chinese, Filipino, South Asian, Japanese, Korean, and Vietnamese Americans (probably self-identification, not further specified)	Data source: 2001 and 2003 California Health Interview Survey (CHIS) (household survey conducted by random-digit dialing with oversampling of areas with high concentrations of specific ethnic groups)	Telephone-administered survey (translated instrument, bilingual interviewers)	Cross-sectional	8192 (foreign-born: Chinese 91%, Filipino 90%,South Asian 96%, Korea 94%, Vietnam 98%)	DV: current smoker vs. non-smoker IV: education, marital status, alcohol consumption, poverty level, health care, insurance, language, length of stay, ethnicity	Logistic regression Stratified by gender, not stratified by nativity	South Asian American women use smokeless tobacco primarily (risk of underestimation of total tobacco use), cross-sectional design, use of existing data constrained investigators’ measures of acculturation (available measures did not capture multidimensional nature of acculturation)
7	Maxwell, Bernaards, McCarthy [62]	2005 in USA	To report smoking rates among different ethnic groups and to compare correlates of smoking among Chinese & Filipino Americans and Hispanic Americans	Chinese & Filipino Americans, Hispanic Americans, additionally African/Black Americans, American Indian/Alaska Natives, Pacific Islanders (probably self-identification, not further specified)	Data source: 2001 California Health Interview Survey (CHIS) (household survey conducted by random-digit dialing with oversampling of areas with high concentrations of specific ethnic groups)	Telephone-administered survey (translated instrument, bilingual interviewers)	Cross-sectional	13,414 (foreign-born: Hispanics 65%, Asians 77%)	DV: current smoker vs. non-smoker IV: age, marital status, education, employment, income, country of birth, years in the USA, level of spoken English	Logistic regression Stratified by nativity and gender	All data are based on self-report, cross-sectional design, only households were reached that had a telephone, only few items were available to assess acculturation, no questions about other tobacco products
8	Rahman, Luong, Divan, Jesser, Golz, Thirumalai, Reedy, Olivas [63]	2005 in USA	To examine smoking prevalence as well as factorsthat may be associated with smoking	Male Vietnamese Americans (probably self-identification, not further specified)	Sample drawn from Vietnamese surnames listed in telephone directory, residential telephone numbers were eligible for sampling, random-digit-dialing sampling procedure	Telephone-administered survey (translated instrument, bilingual interviewers)	Cross-sectional	660 (100% foreign-born)	DV: current smoker vs. non-smoker IV: age, income, education, marital status, English language proficiency, language used at home, language used with friends, length of stay in USA, blood cholesterol check, routine physical check up, binge drinker, multiple sex partners	Logistic regression	Random-digit-dialing methodology excluded potential respondents without telephones, self-report of smoking, findings may not be representative of the behavioural risk factors for Vietnamese who reside in other states than California
9	Wilkinson, Spitz, Strom, Prokhorov, Barcenas, Cao, Saunders, Bondy [64]	2005 in USA	To analyze smoking by age, education, acculturation, and country of birth, to investigate differences in smoking behaviour among US- and foreign-born smokers and to examine role of exposure to US culture in smoking	Mexican Americans (probably self-identification, not further specified)	Data was used from ongoing cohort of Mexican American households (participants were recruited through random-digit dialing, “block walking”, “intercept” (i.e., recruiting individuals from e.g. community centers or local health clinics), and networking via already enrolled participants)	Self-administered survey (translated instruments, bilingual interviewers present)	Cross-sectional	5,030 (70% foreign-born)	DV: current smoker vs. non-smokerIV: age, gender, education, acculturation, age at migration, contextual level (home ownership, Spanish speaking, more than high school education, US-born, median age), age at migration	Logistic regression Stratified by country of birth (1 model for US-born persons, 2 models for Mexican-born persons)	Cross-sectional design, unable to assess influence of family contexts on smoking, smoking was self-reported, other key variables (age at migration and country where smoking was initiated) were calculated on the basis of self-reports (not directly assessed)
10	Hofstetter, Hovell, Lee, Zakarian, Park, Paik, Irvin [65]	2004 in USA	To examine tobacco use and its determinants with special emphasis on acculturation	Korean Americans (probably self-identification, not further specified)	Sampling frame based on residential telephones listed to Korean surnames, list was sorted into random order for interviewing	Telephone-administered survey (translated instrument, bilingual interviewers)	Cross-sectional	2,830 (94% foreign-born)	DV: smoking; smoking uptake; age at first cigarette; smoke cigarette if offered by a friend (yes vs. no) IV: age, education, employment status, acculturation, social support, models who smoke	Ordinary least squares analysis and logistic regression Interaction terms between gender and each IV	Design as telephone survey where no information on persons without residential telephones was available, cross-sectional design
11	Shelley, Fahs, Scheinmann, Swain, Qu, Burton [66]	2004 in USA	To describe tobacco use knowledge, attitudes and behaviours and to examine association between patterns of tobacco use and acculturation	Chinese Americans (self-identification)	List of Chinese surnames, application of stratified systematic sampling procedure (2 stages: first, sample cohort of Chinese American households was identified and data gathered of all adults within households; second, 3 sample groups of adults aged 18-64 years were selected for extended interview: (1) current smokers, (2) nonsmoking men, and (3) women	Interviewer-administered survey (translated instrument, bilingual interviewers)	Cross-sectional	712 (97% foreign-born)	DV: current smoker vs. former smoker, never smoker vs. ever smoker IV: age, education, employment, marital status, insurance, health care use, knowledge of tobacco risks, English language used at home, reading of English newspapers	Logistic regression Highest statistical analysis only for males	Preliminary results to be confirmed by analysis of full sample, sampling frame was based on subjects living in households with listed telephones, self-reports were not validated
12	Fu, Ma, Tu, Siu, Metlay [67]	2003 in USA	To assess whether greater level of acculturation was associated with decreased current cigarette smoking	Chinese Americans (self-identification)	Recruitment in medical practices with fluent Chinese-speaking providers	Self-administered survey (translated instrument, bilingual interviewers present)	Cross-sectional	541 (98% foreign-born)	DV: current smoker vs. non-smoker IV: acculturation Adjusted for age, study site, education level, income	Logistic regression Highest statistical analysis only for males	Language proficiency only one dimension of acculturation, study subjects were a convenient sample of patients at medical or dental practices, differences between participating and non-participating clinics may limit validity of results, self-reported smoking behaviour, very small number of female cigarette smokers
13	Juon, Kim, Han, Ryu, Han [68]	2003 in USA	To examine prevalence of smoking and the correlated factors of smoking	Male Korean immigrants (country of birth)	Community-based sampling in six Korean churches and two Korean grocery stores	Self-administered survey (translated instrument, in exceptional cases interviewer-administered survey with bilingual interviewers)	Cross-sectional	771 (100% foreign-born)	DV: current smoker vs. never smoker; former smoker vs. never smoker IV: age, education, marital status, employment, length of stay, history of hypertension, regular check-up, alcohol use	Logistic regression	Exclusion of those who do not go to church or groceries, underreporting of smoking during survey in church, length of stay only one dimension of acculturation
14	Ma, Shive, Tan, Toubbeh [69]	2002 in USA	To determine tobacco use rates and to determine demographic variables that are potential predictors of tobacco use	Chinese, Korean, Vietnamese and Cambodian Americans (self-identification)	Random selection and division of Asian American community organisations into clusters, stratification of selected organization clusters according to 4 ethnicity groups (Chinese, Korean, Vietnamese, Cambodian), use of proportional allocation procedure in which sample sizes were assigned proportionally to subgroups	Interviewer-administered survey (bilingual interviewers, no information on translated instrument)	Cross-sectional	1,174 (94% foreign-born)	DV: current smoker vs. non-smoker IV: gender, age, education, marital status	Logistic regression Not stratified by nativity	Cross-sectional design, self-report procedure, modifications of the simple random sampling design had to be applied to facilitate greater access to the communities
15	Yu, Chen, Kim, Abdulrahim [70]	2002 in USA	To describe and examine factors significantly associated with smoking	Chinese Americans (self-identification)	List of Chinatown residents was generated by merging compiled surnames, telephone directories and Chinese newspaper subscribers, two-stage probability sampling method to randomly select Chinese households	Interviewer-administered survey (translated instrument, bilingual interviewers)	Cross-sectional	644 (100% foreign-born)	DV: current smoker vs. non-smoker IV: education, usual source of health care, knowledge of cancer warning signs	Logistic regression Highest statistical analysis only for males	No limitations reported Very small number of female smokers, cross-sectional design, selection bias due to sampling procedure, sample was restricted to Chinese men aged 40-69 years
16	Kim, Yu, Chen, Kim, Brintnall, Vance [71]	2000 in USA	To examine smoking behaviour, knowledge and beliefs and to better understand tobacco-related factors	Korean Americans (probably self-identification, not further specified)	Compilation of list with Korean household names, Korean newspaper subscribers, participants in Korean community centre, two-stage probability sampling method to randomly select Korean households	Interviewer-administered survey (translated instrument, bilingual interviewers)	Cross-sectional	263 (100% foreign-born)	DV: current smoker vs. non-smoker IV: age, education, religion, English proficiency, length of stay in USA	Logistic regression Highest statistical analysis only for males	No limitations reported Very small number of female smokers, cross-sectional design, selection bias due to sampling procedure, sample was restricted to Korean men aged 40-69 years
17	Shankar, Gutierrez-Mohamed, Alberg [72]	2000 in USA	To describe smoking prevalence and to evaluate attitudes and beliefs towards smoking	El Salvadoran immigrants (country of birth)	Survey of Salvadoreans living in the Washington DC metropolitan area (not further specified, with reference to publication on sampling details)	Interviewer-administered survey (bilingual interviewers, no information on translated instrument)	Cross-sectional	1,458 (100% foreign-born)	DV: prevalence difference (current vs. never; former vs. never) IV: age, gender, marital status, household size, employment status, income, years of schooling, age at migration	Linear regression	No limitations reported Very small number of female smokers, recently immigrated group and heavily weighted towards young persons, no association between smoking and socioeconomic status were found at all
18	King, Polednak, Bendel, Hovey [73]	1999 in USA	To examine differences in smoking between foreign- and native-born persons and to examine impact of demographic and socioeconomic status on smoking	African/Black Americans (country of birth, self-identification)	Data source: National Health Interview Survey (NHIS) (nationally representative survey in the USA conducted annually), additional merging of Cancer Control Supplement (CCS) and Cancer Epidemiology Supplement (CES) to increase representation of African/Black Americans	Interviewer-administered survey (no information on translated instrument or bilingual interviewers)	Cross-sectional	16,738 (7% foreign-born)	DV: current smoker vs. non-smoker IV: age, gender, education, income, length of stay in USA, nativity (native- vs. foreign-born), employment, marital status, region	Logistic regression	Possibility of underestimating smoking prevalence due to undocumented residents and non-respondent bias, sampling error due to undercoverage, NHIS data is cross-sectional
19	Wiecha, Lee, Hodgkins [74]	1998 in USA	To measure prevalence and patterns of tobacco use and to identify smoking risk factors and readiness to quit smoking	Male Vietnamese American (probably self-identification, not further specified)	Vietnamese names were used to construct a search list, phone numbers of persons with one of these names were obtained by manual abstraction of directories representing communities with the largest Vietnamese populations	Telephone-administered survey (bilingual interviewers, no information on translated instruments)	Cross-sectional	774 (100% foreign-born)	DV: current smoker vs. non-smoker IV: age, smoking parents, education, exercise, depression, health insurance, part of Vietnam raised in	Logistic regression	Restriction to males only, sampling frame was based on subjects living in households with listed telephones, depression measures used are likely to have been relatively imprecise
20	Hyman, Fenta, Noh [75]	2008 in Canada	To present data on risk and protective factors associated with smoking	Ethiopian immigrants (country of birth)	Snowball technique (membership lists of Ethiopian organizations) and list of Ethiopian names was compiled using city telephone directory as sampling frame, household selection using simple random sampling method, additional public relations work	Interviewer-administered survey (translated instrument, bilingual interviewers)	Cross-sectional	342 (100% foreign-born)	DV: current smoker vs. non-smoker IV: age, marital status, importance of religion, education, employment status, length of stay in Canada, exposure to pre-migration trauma, refugee camp internment, number of post-migration life events, satisfaction with social support	Logistic regression Results presented from bivariate regression (multivariate analysis carried out only for males – results not presented) Bivariate analysis stratified by gender	Exclusion of potential candidates if they had no telephone, stable address or membership status in Ethiopian organisations, small number of female smokers prevented further statistical analyses
21	Kabir, Clarke, Keogan, Currie, Zatonski, Clancy [76]	2008 in Ireland	To identify significant predictors of smoking	Polish immigrants (country of birth)	Advertisement in Polish lifestyle magazine, 10 Polish interviewers were posted at busy intersection of the Dublin city area (with numerous Polish shops)	Interviewer-administered survey (translated instrument, bilingual interviewer)	Cross-sectional	1,545 (100% foreign-born)	DV: current smoker vs. non-smoker IV: education, employment, duration of stay Adjusted analysis (variables not stated)	Logistic regression	Very constrained generalisability of study findings (high risk of selection bias due to sampling procedure)
22	Reiss, Spallek, Razum [77]	2010 in Germany	To analyse whether smoking differs between groups with increasing duration of stay	Ethnic German immigrants from Former Soviet Union countries (birth in Germany, citizenship, naturalization)	Data source: German microcensus (annual countrywide census including 1% of all German households, participation in survey is obligatory)	Interviewer-administered survey (no information on translated instrument or bilingual interviewers)	Cross-sectional	13,158 (100% foreign-born)	Smoking prevalence with different lengths of stay (3 categories)	Descriptive analysis Chi-square-test Analysis stratified by gender, age and education	Cross-section design, very small number of smokers aged 65 years and older, since survey is carried out on household level, ‘cluster effect’ cannot be ruled out
23	Reeske, Spallek, RazumGermany [78]	2009 in	To investigate smoking patterns among groups with increasing duration of stay and among native-born persons	Turkish immigrants (birth in Germany, citizenship, naturalization + information on parents)	Data source: German microcensus (annual countrywide census including 1% of all German households, participation in survey is obligatory)	Interviewer-administered survey (translated instrument, no information on bilingual interviewers)	Cross-sectional	12,288 (59% foreign-born)	Smoking prevalence among first- and second-generation immigrants and with different lengths of stay among first-generation immigrants (3 categories)	Descriptive analysis Chi-square-test Analysis stratified by gender, age and education	Cross-section design, partially very small number of smokers after stratification, since survey is carried out on household level, ‘cluster effect’ cannot be ruled out
24	Nierkens, de Vries, Stronks [79]	2006 in the Netherlands	To assess smoking prevalence and its socioeconomic gradients among three immigrant populations	Turkish, Moroccan, Surinamese immigrants (country of birth and parents’ country of birth)	Data sources: (1) SUNSET study (Surinamese in the Netherlands: Study on Ethnicity and Health), general healh questionnaire carried out by the Munisipal Health Organisation Amsterdam (population surveys where samples were drawn from municipal population register)	Interviewer-administered survey (translated instrument, bilingual interviewer)	Cross-sectional	1,773 (100% foreign-born)	Percentage of current smokers and former smokers by gender, age and education	Descriptive analysis Percentages with 95% confidence intervals and Odds Ratios	Cross-section design, self-reported smoking status, no figures about exact response rates of Turkish and Moroccan sample (selection bias cannot be ruled out)
25	Vedøy [80]	2013 in Norway	To investigate the association between education and smoking status and to examine if associations fit the pattern predicted by the model of the cigarette epidemic	Turkish, Iranian, Pakistani, Vietnamese, Sri Lankan immigrants (country of birth)	Data source: HUBRO (Oslo Health Study) and Immigrant-HUBRO (population surveys where samples were drawn from municipal population register; citizens born in 1940, 1941, 1955, 1960 and 1970)	Self-administered survey (translated instrument, no information on presence of bilingual interviewers)	Cross-sectional	4,060 (100% foreign-born)	DV: current smoker vs. non-smoker IV: country of birth, age, education, marital status	Logistic regression Analyses stratified by gender	Cross-section design, self-reported smoking status, low response rates among immigrants (selection bias cannot be ruled out), selection caused by variations in immigration history might have influenced differences between immigrant groups
26	White, Harland, Bhopal, Unwin, Alberti [81]	2001 in UK	To present representative data on smoking	Chinese immigrants (probably self-identification, not further specified)	First, name analysis of Health Services register; second, publicity aimed at Chinese community; third, respondents identified other Chinese residents known to them	Party self-administered, partly interviewer-administered survey (translated instrument, bilingual interviewer)	Cross-sectional	380 (100% foreign-born)	Percentage of current, ex, and never smokers by gender, age, social class, marital status	Descriptive analysis Chi-square-test, Mantel-Haenszel test Age-standardisation (direct method)	Due to the used methodology impossible to provide an accurate response rate, findings may not be representative for Chinese who reside in other areas of the UK, very small sample size, more detailed information needed on types and daily patterns, knowledge and attitude towards smoking
27	Girgis, Adily, Velasco, Garden, Zwar, Jalaludin, Ward [82]	2009 in Australia	To determine associations of tobacco use and tobacco control indicators	Arab immigrants (probably self-identification, not further specified)	Recruitment of Arab immigrants (18-65 years) via Arab speaking general practitioners	Self-administered survey (translated instrument, no information on presence of bilingual interviewers)	Cross-sectional	1,371 (100% foreign-born)	DV: current smoker vs. non-smoker, recall of cessation advice vs. no recall, nicotine dependence (scale), readiness to quit (scale) IV: age, gender, education, employment, marital status, health status	Logistic regressions & linear regressions	Selection bias due to sampling procedure via general practitioners (low response rate), self-reported tobacco use

Concerning the sampling procedures of the studies generating primary data (19 of 27), the vast majority used a list-based (mainly a telephone list-based) technique with focus on names specific to the immigrant group or a community-orientated (via migrant organisations or networks) sampling strategy. It has to be noted that (I) a list-based approach is restricted to only those appearing on, for example, the telephone list, and (II) a random sample for a quantitative study can hardly be realised by using a community-orientated approach as it is highly likely to include predominantly socially integrated people.

A major part of the studies (18 of 27) applied both translated instruments and bilingual interviewers. Six studies reported either the use of translated instruments or the employment of bilingual interviewers. Two studies did not provide any information in this regard and one study was conducted in the host country language (English) only. Applying translated, culturally adapted, and validated instruments – especially on acculturation and smoking – as well as bilingual interviewers may lower the risk of selection and information bias [46,47]. If, however, the risk of selection bias is high, the generalisability of the study results to a group other than the selected one is questionable (see Table 4).

Moreover, using self-reported data to assess smoking behaviour may lead to an under- or overestimation of the smoking status, for example, due to socially desirable responses. As all studies used self-reported data, information bias cannot be excluded. However, one study validated the self-reported data by using expired carbon monoxide measures and showed a significantly high correlation between self-reports and measurements. Furthermore, all studies applied a cross-sectional design. Here, factors associated with smoking and smoking status are measured simultaneously. Thus, it is neither possible to assess causal pathways between factors associated with smoking and smoking status nor to draw conclusions on changes over time. Additionally, as the sample in cross-sectional studies comprises different age or birth cohorts, the study findings may also be the result of a cohort effect.

## Discussion

Our analysis shows that smoking among immigrants from economically developing non-western to economically developed western countries is positively associated with (I) a low acculturation level among men, (II) a high acculturation level among women – as measured by acculturation scales or proxy measures such as language proficiency or duration of stay – and in general (III) with being male, of younger age, non-married, and employed. Smoking was also associated with a low educational level among men and a high education among women.

Socio-economic and socio-demographic factors such as marital status, education, employment status, age, and gender are already known to determine smoking in any population group, irrespective of their migration status. Among immigrants, acculturation is an additional factor linked to smoking. One possible explanation can be found in the transition brought about by the immigration from non-western to western countries, which occupy different positions in the ‘smoking epidemic’. Western countries tend to be located towards the advanced phases of the epidemic, whereas non-western countries tend to be located more towards the early phases, and smoking among women tends to lag behind that in men [48,17]. This is supported by higher prevalences among men from non-western countries compared to men from western countries, lower prevalences among women from non-western countries compared to women from western countries and by the large gap in smoking between men and women from non-western countries as seen in Table 1. It is compatible with the hypothesis that with the immigration from non-western to western countries immigrant men and women may ‘import’ their smoking behaviour from the country of origin to the host country. If acculturation is regarded as process which starts with the immigration to the host country, this might explain the high smoking prevalence among less acculturated men and the low smoking prevalence among less acculturated women. Furthermore, with increasing duration of stay in the host country, immigrants might ‘move’ towards the advanced phases of the smoking epidemic and adapt to the smoking behaviour of the host country. This is likely to be the result of an ongoing acculturation process. In many non-western countries smoking is still uncommon and socially unacceptable among women but highly acceptable among men [42-45]. This pattern of social support of smoking might account for the smoking behaviour among recent immigrants. In western societies smoking is equally accepted among men and women [42-45]. Immigrants might identify with these values and attitudes and adopt them in the course of the acculturation process towards a higher smoking prevalence among women and a lower one among men. These findings correspond well to the ‘operant model of acculturation’ as proposed by Landrine & Klonoff [49]. It predicts that health behaviours with a high prevalence among initially low-acculturated immigrants will decrease in prevalence with increasing acculturation, whereas health behaviours with a low prevalence among low-acculturated immigrants will become more common with advancing acculturation. This model has already been successfully applied to health behaviours such as smoking, diet, alcohol use, and exercise [49,50]. The phenomenon that higher levels of acculturation are associated with increases in unfavourable health behaviours is also known as the ‘immigrant paradox’.

Only few studies so far have explicitly focused on the relationship between acculturation and the stages of the ‘smoking epidemic’; the studies investigating the course of the epidemic among immigrants have mainly focused on its association with educational level [51,52]. In general, research on smoking behaviour among immigrants with focus on acculturation is in its infancy in Europe. Only one of the European studies included in this review explicitly stated that it also measured acculturation.

However, this should not divert from the fact that, besides acculturation, there are several other determinants of smoking on the individual, cultural, economic and political level. There might also be interactions between certain determinants (e.g. between education and employment status). Differences between countries of origin and host countries regarding, for example, cigarette prices or bans on smoking in public/work places might additionally affect smoking in immigrants.

### Strengths and limitations of the review

This review combines the concept of acculturation and the model of the ‘smoking epidemic’ for the research on smoking in immigrants. So far, studies focused on either of both aspects. Linking the health transition in terms of the ‘smoking epidemic’ to the acculturation process is the major strength of this review.

Besides this strength, the review has some limitations: first, factors associated with smoking were dichotomised only (e.g. high vs. low, long vs. short – see Table 2). This was done in order to better illustrate the main findings. Such a simplification leads to a loss of information and to a possible misclassification as specific definitions and measurements of the variables (e.g. long vs. short length of stay) are likely to vary between the studies. Second, 19 of 27 studies were conducted in the USA and most focused on Asian immigrants. This might bias the findings of the review towards this immigrant group or towards US immigrants in general. At the same time it reveals the need to further investigate factors associated with smoking among immigrants in countries other than the USA. Third, the retrieval procedure was performed by using the database PubMed only. Thus, it cannot be excluded that additional articles on the topic under study appeared in journals not listed in the database. However, as the database covers a large number of journals from the life sciences and biomedicine, it is not likely that a substantial number of papers on smoking behaviour among immigrants have been missed.

## Conclusion

### Implications for future research

While 11 of 27 studies stratified their analysis by gender, seven studies only adjusted for gender. Future studies should not only appreciate gender differences in smoking behaviour by adjusting for gender, but explicitly assess gender as a potential modifying variable. This might also help to further explore the ‘immigrant paradox’ where higher acculturated women are more likely to participate in unhealthy behaviours. Moreover, measures of acculturation should not just be unidimensional, with either a weak or a strong orientation towards the culture of the host country. As immigrants do not have to give up their traditional culture in the process of acculturation, unidimensional measures do not satisfactorily reflect the inherent multidimensionality of acculturation. Such multi-item scales should furthermore be based on theoretical or conceptual work. This could promote a more *direct* approach to studying the process of acculturation (rather than relying on proxy measures) and thus help to better understand the association between acculturation and health behaviour. Additionally, information bias might be minimised.

All studies we could include in this review were cross-sectional. As acculturation is a *process*, only longitudinal studies will properly track how changes in acculturation affect health-related behaviour over time. This also applies to other variables which may have an effect on smoking such as education or economic factors; they are time-variant as well and therefore prone to change with increasing duration of stay in the host country. Besides, not only the situation in the host country influences the smoking behaviour among immigrants, the process of immigration itself and the situation in the countries of origin – and thus the context of migration – may have an impact on health behaviour. Consequently, future studies should focus on a *life-course* perspective to reveal mechanisms and determinants responsible for uptake, maintenance and cessation of smoking [53,54].

### Implications for prevention and health promotion

Health professionals need to be aware of the patterns of smoking among immigrants identified in this review and adapt existing health promotion programs or plan and initiate new programs accordingly. In particular, they need to employ different strategies for immigrant men and women. Programs for male immigrants should aim to further decrease smoking prevalence, whereas programs addressing immigrant women should aim at preventing smoking initiation. Immigrants of both sexes need to be made aware of the social and cultural forces that operate during the process of acculturation and may affect their smoking behaviour. As smoking and acculturation are not only individual but also social and group phenomena, key persons from immigrant communities should be involved in implementing strategies in their communities, settings, and networks [55]. Thus, knowing the factors associated with smoking among immigrants is only the first step towards its prevention.
